# Neurocysticercosis in non-endemic regions: The experience of Qatar

**DOI:** 10.3389/fneur.2023.1173909

**Published:** 2023-04-20

**Authors:** Naim Haddad, Yanal Shaheen, Mohammed Abunaib, Gayane Melikyan, Ahmed El Sotouhy, Farah Wahbeh, Areej Nauman, Fatima Al-Maadid, Mohamed Soliman, Boulenouar Mesraoua, Hisham Elkhider, Ziyad Mahfoud

**Affiliations:** ^1^Department of Neurology, Weill Cornell Medicine-Qatar, Ar-Rayyan, Qatar; ^2^Department of Neuroradiology, Hamad Medical Corporation, Doha, Qatar; ^3^Department of Population Health Sciences, Weill Cornell Medicine-Qatar, Ar-Rayyan, Qatar

**Keywords:** neurocysticercosis, Qatar, seizures, epilepsy, calcified granuloma, antiseizure drugs, antihelminthic treatment

## Abstract

**Objective:**

To describe the occurrence and features of Neurocystircercosis (NCC) in Qatar.

**Background:**

Qatar has a mixed population of natives and expats. NCC is not endemic to the region, but clinical practice suggests its occurrence in large numbers.

**Design/ methods:**

A database was created to summarize information retrospectively collected on patients with NCC seen through the national health system (HMC) between 2013 and 2018. We identified demographic and disease related variables (clinical manifestations, investigative findings, treatment and outcome) for all patients.

**Results:**

Out of 420 identified NCC patients, 393 (93.6%) were men, and 98.3% were immigrants from NCC endemic countries such as Nepal (63.8%) and India (29.5%). Eighty percent of patients presented with seizures, with the majority (69%) experiencing generalized tonic–clonic seizures. Five percent presented with status epilepticus. Headaches, the second most common complaint, were reported in 18% of subjects. On imaging, 50% had a single lesion while 63% included pathology at the calcified stage. The lesions were parenchymal in 99.5% of cases, predominantly in the frontal lobe (59%). Thirteen percent were diagnosed incidentally on imaging, mainly in the form of isolated calcified non enhancing lesions. Albendazole was received by 55% of patients, and phenytoin was the most prescribed anti-seizure drug (57%). When long term follow up was available, 70% of the patients presenting with seizures were completely seizure free.

**Conclusion:**

NCC is prevalent in Qatar, mainly within the large Southeast Asian immigrant population. NCC is currently a significant contributor to the epilepsy burden in Qatar, often with a good outcome regarding seizure control. NCC with intraparenchymal single lesion shares a large proportion of our cohort.

## Introduction

Neurocysticercosis (NCC) is the most common central nervous system (CNS) parasitic infection in humans and is an important cause of acquired epilepsy around the world (1–5). Areas in which NCC is endemic include Latin America, sub-Saharan Africa, the Indian Subcontinent and Southeast Asia. However, an increasing number of cases are surfacing in nonendemic regions most likely due to migration and travel (1–6). NCC is acquired *via* the fecal-oral route when humans ingest *Taenia solium* tapeworm eggs (1–5). Following ingestion, *Taenia* eggs develop into larvae referred to as cysticerci which are fluid-filled cysts that lodge in soft tissues such as the brain, and typically evolve over years (1–4). The most common clinical manifestation of NCC is seizures (1–5), (7–10). NCC can also cause increased intracranial pressure which results in a variety of symptoms including headache, nausea, and vomiting (1–5).

Qatar is a small country in the Arabian Peninsula, with a mixed population of natives and expats. NCC is not endemic to the region (4, 6), but clinical practice suggests its occurrence in increasing numbers. This report aims to describe the occurrence and features of NCC in Qatar.

## Methods

Data was retrospectively collected on patients with definitive or probable NCC seen through the national health system at Hamad Medical Corporation (HMC) between 2013 and 2018. HMC hosts the main adult comprehensive neurology services in the country. Definitive NCC was mainly based on visualizing the scolex on imaging, or much less often on histology. Probable NCC had highly suggestive lesions on imaging with congruent clinical manifestations, typically with history of residence or travel in endemic regions (11, 12). After Institutional Review Board approval, demographic and disease-related variables (clinical manifestations, radiological/EEG findings, treatment, and outcomes) were identified for all patients. A database was created to summarize the gathered information. In the statistical analysis, the demographic and clinical characteristics of patients were described as mean and standard deviation for numerical variables and as frequency distribution for categorical variables. All analyses are carried out using IBM-SPSS (version 27, Armonk NY, United States).

## Results

### Demographics

This study included 420 patients with a mean age (years) of 31.8 ± 9.5. Three hundred ninety three (93.6%) were male, and 27 (6.4%) were female. With regards to nationality, the majority of patients were Nepalese (268; 63.8%), and Indian (124, 29.5%). Other nationalities included Bangladeshi, Sri Lankan, Pakistani, Thai, Filipino, Qatari, North Korean, Congolese, Djiboutians, Egyptian, South African, and Ethiopian. Over 98% of patients were immigrants from endemic countries, and only 1.2% were local Qataris (Table 1).

**Table 1:** Demographic characteristics.

Total number of patients	420
Age (mean ± SD)	31.8 ± 9.5 years
Male	393 (93.6%)
Female	27 (6.4%)
Nationality	Nepalese –268 (63.8%)
	Indian –124 (29.5%)
	Qatari –5 (1.2%)
	Other –23 (5.5%)

### Seizure types and other clinical manifestations

Three hundred thirty-five (80%) patients presented with seizures. Among these patients, 69% experienced generalized tonic–clonic seizures by description. The other less common seizure types were focal aware motor seizures (9.6%), focal aware nonmotor seizures (1.5%), and focal impaired awareness seizures (4.2%). 22% of subjects reported more than one seizure type. Additionally, 5% of patients experienced status epilepticus.

Other clinical manifestations included headache (18%), nausea and vomiting (3.8%), focal neurologic deficits (3.8%), and altered mental status (3.6%). The less common presentations included cranial nerve (CN) palsy which was experienced by one patient, and papilledema and intracranial hypertension (IIH) which were experienced by four patients. Fifty-four patients (13%) were diagnosed incidentally on imaging, mainly in the form of isolated calcified non enhancing lesions (Table 2).

**Table 2:** Clinical manifestations.

Seizures	335 (80%)
Generalized tonic–clonic	232 (69%)
Focal aware, motor	32 (9.6%)
Focal impaired awareness	14 (4.2%)
Focal aware, nonmotor	5 (1.5%)
Unclassified seizure (other)	71 (21.2%)
Status epilepticus	17 (5%)
Loss of consciousness otherwise unspecified	11 (2.6%)
Headache	77 (18%)
Nausea/vomiting	16 (3.8%)
Focal neurologic deficits	16 (3.8%)
Altered mental status	15 (3.6%)
Increased intracranial pressure/Papilledema	4 (1.0%)
Cranial nerve palsy	1 (0.2%)
Incidental	54 (12.9%)
Other	30 (7.1%)

### Diagnostic features

*Radiological findings*: Four hundred seven (97%) patients underwent computed tomography (CT) scans, and 196 (47%) underwent magnetic resonance imaging (MRI). Approximately 53% of patients had only CT scans, 3.1% had only MRIs, and 43.6% had both. All patients were classified in terms of the number of lesions, stages and location. A single lesion was seen in 50% of patients, two lesions in 18%, and three or more lesions in 32%. With regards to the staging of NCC lesions, 7.4% of cases had cysts at the vesicular stage, 49.5% had degenerating cysts (colloid and/or granular/nodular stages), and 63% had calcified lesions. Thus, almost 20% had lesions at different stages concomitantly. The majority of patients (418, 99.5%) had parenchymal lesions. Only six patients had ventricular cysts, and four had subarachnoid ones. We did not identify any cases with spinal cysts. Intraparenchymal lesions were found in the frontal lobe in 59% of cases, in the temporal lobe in 25%, in the parieto-occipital lobes in 59%, in deep cerebral structures in 11% and in the brainstem/cerebellum in 4% (Table 3).

**Table 3:** Radiological findings.

*Number of lesions*	
Single	211 (50%)
Two	76 (18%)
≥ three	133 (32%)
*Staging of NCC*	
Calcified	264 (63%)
Colloid and/or granular/nodular	208 (49.5%)
Vesicular	31 (7.4%)
*Location of NCC*	
Parenchymal	418 (99.5%)
Frontal lobe	249 (59%)
Parieto-occipital lobes	248 (59%)
Temporal lobe	105 (25%)
Deep cerebral	45 (11%)
Posterior fossa	16 (4%)
Subarachnoid	4 (1%)
Ventricular	6 (1.4%)

*EEG findings*: Thirty-eight (9%) patients underwent EEGs. Among them, only nine patients had abnormal findings with only two cases showing epileptiform discharges (spikes) while the remaining seven had focal or generalized slowing.

### Treatment and outcome

Albendazole was the antiparasitic agent of choice, received by 55% of patients, with 49% also receiving dexamethasone. Only three patients underwent neurosurgical procedures.

Phenytoin is the most used anti-seizure medication (ASM), given in 57% of cases, followed by levetiracetam in 14%, valproate and carbamazepine in 3% each. Of the patients with seizures, 90% were on a single ASM, 4% were on two or more ASMs, and 6% were not taking any ASMs on their last documented note.

With regards to outcome, 119 (28%) patients had a clinic follow up beyond 6 months from the initial diagnosis. Approximately half of them, 59 subjects, underwent head reimaging. Of the 103 patients who initially presented with seizures and had reliable follow up, 72 (70%) were completely seizure free as per the last documented clinic note.

The majority of reimaged patients (51%) underwent MRI, 39% underwent CT, and 10% underwent both MRI and CT. The NCC disappeared on reimaging in only 12% of subjects, with 80% showing either an unaltered appearance or just partial improvement without complete resolution.

## Discussion

In the current study, we report on more than 400 patients with NCC in a country (Qatar) where the disease was historically nonexistent due to the absence of swine farming. This is by far the largest reported series from the Middle East and Arabian Peninsula (13–16). The infection is likely to have been acquired abroad as most patients are immigrants from endemic countries, notably Nepal and India. This trend is also reported in North American series, where the majority of patients are immigrants from Central and South America with the onset of symptoms occurring years after immigration (17–21). The predominance of young male patients in our database can be attributed to the disproportionately large southeast Asian male population living in Qatar (Planning and Statistics Authority), but other case series from the United States have also noted a male to female ratio of up to 1.5–2:1 (17, 18). Similar patterns of sex distribution for NCC are present in some reports from endemic countries as well; a retrospective analysis showed that 71.4% of NCC patients in Nepal were male (22).

Not surprisingly, 80% of patients in our cohort presented with seizures. This is consistent with the current knowledge that seizures are the predominant manifestation of NCC (1–5), (7–10). Previous reports from Qatar alluded to the contribution of NCC to the overall seizure burden. In a study by Pathan et al. investigating the computed tomography abnormalities in patients presenting with first seizure to the emergency department in Qatar, the most common ones consisted of frank neurocysticercosis (9.2%) and calcified (6%) lesions (23). In another study mapping epilepsy in Qatar by Haddad et al., the authors found that NCC was the etiology for epilepsy in 10% of subjects of Southeast Asian background (24).

On brain imaging, 50% had a single lesion while 63% included lesions at the calcified stage. The lesions were parenchymal in 99.5% of cases, predominantly in the frontal lobe (59%). The abundance of single lesion cases in our cohort is reminiscent of described case series coming from Nepal and India, the countries of origin for most of our NCC cases. In Nepal, single lesions were seen in 62% of patients (22). A study from India also found that 64% of patients with probable NCC had single lesions (25). Many more reports suggest that single granulomas are more frequent in India compared to Latin America (5, 26). The paucity of extraparenchymal lesions in our cohort follows the same explanation. In Indian series, subarachnoid and ventricular localizations are less frequently identified than in Latin American series (5, 26, 27). Such heterogeneity within NCC is noted in non-endemic countries depending on the native geographical background of the patient population. North American series contain a higher rate of subarachnoid and ventricular NCC compared to ours (17–19, 21), again reflecting the Latin American background where the likely distant infestation occurred in many of their patients. We also found that 13% of our subjects were diagnosed incidentally on imaging, mainly in the form of isolated calcified non enhancing lesions. NCC can be asymptomatic (1, 5, 28). This should be recognized in non-endemic areas also; in a report of 48 patients diagnosed in Kuwait from 2014 to 2019, nine (18.7%) presented no apparent related symptoms (16).

Even though 80% of our NCC patients presented with seizures, EEGs were performed in less than 10% of subjects. The treating physicians were apparently comfortable with a diagnosis of seizures based on the clinical description and the correlated etiology on brain imaging. Only two out of 39 EEG studies showed epileptiform discharges. This experience, albeit limited, suggests the routine EEG to be clearly lacking in sensitivity in the context of NCC and seizures. In the existing literature, the rate of EEG interictal epileptiform discharges vary widely across the different publications, ranging from 6 to 75% of patients with NCC (29). Spikes are more likely to be detected in patients with enhancing lesions or viable cysts compared to patients with pure calcifications (29, 30).

As for treatment choices, Albendazole was received by 55% of patients, which roughly corresponds to the rate of NCC subjects with other than pure calcifications on imaging. According to the latest reviews and guidelines, antihelmintic therapy is recommended for potentially viable parenchymal NCC. Albendazole alone or in combination with Praziquantel seem to enhance the destruction of parasitic cysts and may lead to fewer seizure recurrences (31–34). Phenytoin was the most prescribed ASM (57%). This choice over newer ASMs is best explained by affordability and global availability, with most of our patients being immigrant laborers on limited income. The current literature does not offer any strong evidence for the choice of ASM in the context of NCC. We found a single open label study comparing carbamazepine and levetiracetam; although there was a trend for better seizure control with carbamazepine, patients treated with the later experienced a higher rate of drug related side effects (35). There is no reliable evidence regarding the duration of ASM treatment either, with the persistence of calcified lesions predicting a higher risk for seizure recurrence (1, 32, 36). We need larger multicenter, randomized, and controlled trials to address the questions of choice and duration of ASMs.

Outcome was only measurable in a fraction of our subjects. Most of our patients were immigrants on short term contracts, and thus returned to their home countries and were lost to follow up. The overall seizure outcome was favorable in the majority, with 70% noted to be seizure free on the last clinic visit. This is compatible with the reported NCC literature. In a study from a rural community in South India, 80% of NCC subjects achieved seizure freedom for more than 2 years. The remaining patients had rather infrequent seizures (37). Goyal et al. reported on 200 patients with epilepsy due to NCC, who were followed for a minimum of 1 year. Only 17 patients (8.5%) had recurrence of their seizures, including 13 (6.5%) who fulfilled the ILAE criteria for drug resistant epilepsy (38, 39). Seizure freedom was often achieved with antiseizure monotherapy (37, 38). Furthermore, amongst 512 patients with intractable epilepsy in Brazil where NCC is endemic, isolated NCC was found in only eight patients (1.56%) (40). Thus, despite the high contribution of NCC to the burden of epilepsy especially in endemic countries, NCC only rarely leads to intractable epilepsy.

Our study has some limitations. It is a retrospective chart review, and more than half of our subjects lacked long term follow up. We do not have serological antibody testing at our disposition to support the NCC diagnosis. However, the enzyme linked immunotransfer blot (EITB), despite being the serological test of choice, has a poor sensitivity in patients with single parenchymal or with only calcified NCC (32). Such patients make up a large proportion of our series and thus would have not benefited much from such testing.

This is by far the largest NCC series from our region, the Middle East and the Arabian Peninsula. NCC is clearly prevalent in Qatar and is predominantly noted in the Southeast Asian immigrant population. NCC is a significant contributor to the epilepsy burden in Qatar, often with a good outcome regarding seizure control. NCC with intraparenchymal single lesion seems to be a common radiological presentation in our cohort. Physicians, especially neurologists and emergency practitioners, should be fully aware of its presence and complications.

## Data availability statement

The original contributions presented in the study are included in the article/supplementary material, further inquiries can be directed to the corresponding author.

## Ethics statement

The studies involving human participants were reviewed and approved by Hamad Medical Corporation -Medical Research Center. Written informed consent for participation was not required for this study in accordance with the national legislation and the institutional requirements.

## Author contributions

NH, GM, AE, BM, HE, and ZM contributed to the conception and design of the study. YS, MA, FA-M, and MS organized the database. NH, YS, and ZM performed the statistical analysis. NH, YS, AN, FW, BM, and ZM wrote the first draft of the manuscript. All authors contributed and approved the final manuscript version.

## Conflict of interest

The authors declare that the research was conducted in the absence of any commercial or financial relationships that could be construed as a potential conflict of interest.

## Publisher’s note

All claims expressed in this article are solely those of the authors and do not necessarily represent those of their affiliated organizations, or those of the publisher, the editors and the reviewers. Any product that may be evaluated in this article, or claim that may be made by its manufacturer, is not guaranteed or endorsed by the publisher.
